# Yoga, *Ahimsa* and Consuming Animals: UK Yoga Teachers’ Beliefs about Farmed Animals and Attitudes to Plant-Based Diets

**DOI:** 10.3390/ani10030480

**Published:** 2020-03-13

**Authors:** Jenny L. Mace, Steven P. McCulloch

**Affiliations:** Centre for Animal Welfare, The University of Winchester, Sparkford Road, Winchester SO22 4NR, UK; Steven.McCulloch@winchester.ac.uk

**Keywords:** *ahimsa*, Buddhism, consuming animals, Hinduism, Jainism, plant-based diet, spirituality, veganism, vegetarianism, yoga

## Abstract

**Simple Summary:**

Yoga is a holistic discipline originating in ancient India. Yoga has links with Hinduism, Buddhism and Jainism based on a shared philosophical framework of unity with all beings and belief in *ahimsa*, meaning non-harming. There is debate in the international yoga community about the spiritual, ethical and health-related links between yoga and plant-based diets. This questionnaire-based research investigates the beliefs about the moral status of farmed animals and attitudes towards plant-based diets of UK yoga teachers. The research found that: (i) UK yoga teachers have very progressive beliefs about farmed animals; (ii) around 30% of UK yoga teachers follow a plant-based diet, which is 25 times the proportion in the general UK population; (iii) nearly 75% desire to follow a plant-based diet; (iv) over two thirds regard plant-based diets as best aligned to their yogic practice; and (v) UK yoga teachers with more progressive beliefs about farmed animals and greater knowledge of agriculture abstain from consuming animal products to a greater extent. The high proportions of UK yoga teachers following vegetarian and plant-based diets, relative to the wider population, are likely based on applying the principle of *ahimsa*, or non-harming, to farmed animals and abstaining from consuming their products.

**Abstract:**

Yoga is a holistic discipline originating in ancient India. Yoga has links with Hinduism, Buddhism and Jainism based on a shared philosophical framework of unity with all beings and belief in *ahimsa*, meaning non-harming. There is debate in the international yoga community about the spiritual, ethical and health-related links between yoga and plant-based diets. This mixed methodology research investigates the beliefs about the moral status of farmed animals and attitudes towards plant-based diets of UK yoga teachers. A sequential mixed-methods design employing a questionnaire and semi-structured interviews is used. This paper focuses on the questionnaire-based phase of the research. Key results are: (i) UK yoga teachers have very progressive beliefs about the moral status of farmed animals; (ii) 29.6% of UK yoga teachers follow a plant-based diet (n = 446), which is 25-fold the proportion in the wider UK population; (iii) 73.9% desire to follow a plant-based diet; (iv) 68.6% regard plant-based diets as best aligned to their yogic practice; and (v) UK yoga teachers with more progressive beliefs about farmed animals and with more self-reported knowledge of agriculture abstain from consuming animal products to a greater extent. The far higher proportions of UK yoga teachers following vegetarian and plant-based diets, relative to the wider population, are likely based on applying yogic teachings such as the principle of *ahimsa* through abstaining from the consumption of animal products.

## 1. Introduction

Yoga is a physical, mental and spiritual discipline originating from ancient India. The *Rig Veda* (c. 1500 BCE) is the earliest known written source of the word *yoga* [1]. Yoga is first defined in later Vedic texts as the ‘firm restraint of the senses’ (6.11) in the *Katha Upanishad* (c. 200 BCE) and as the ‘separation from contact with suffering’ (6.23) in the *Bhagavad Gita* (c. 200 BCE). 

Yoga is a key component of Hinduism, which is conservatively estimated to originate from c. 1500–1200 BCE. Yoga also forms a key component of Buddhism (originating from c. 600 BCE) and Jainism, which is thought to have begun emerging in c. 500–100 BCE [1,2]. Hinduism, Buddhism and Jainism all share a cultural history. They all teach *ahimsa*, meaning non-harm to all beings. The meaning of *ahimsa* stems from its constituent parts: ‘*a*’ meaning absence and ‘*hims*’ stemming from ‘*han*’ meaning to harm, injure or kill [3]. 

The text *Patañjali’s Yoga Sūtra* (c. 400 CE) is considered the first systematic representation of yoga. This text is typically seen as a Hindu text but with major influences from Buddhism [4]. In the 12^th^ century, yoga was first documented as a *darsana* (a philosophical system) of both orthodox and heterodox Indic traditions. It subsequently became one of the official six schools of Hinduism [1]. *Hatha* yoga, influenced by Buddhist tantric philosophy, emerged in the medieval period as a branch of yoga emphasising physical practices. This evolution of yoga is encapsulated in its name as ‘*hatha*’ can translate as force [1]. 

Modern hatha yoga in the west has become synonymous with ‘yoga’ and is practiced primarily for enhancing health and relaxation, generally as part of the modern day ‘holistic milieu’ and often with a ‘New Age’ orientation [5,6]. Some religious scholars argue that yoga is not a monolithic Hindu or Indian tradition [7]. It is rare for modern western yoga teachers and practitioners to identify as Hindu, Buddhist or Jain. They may or may not identify as spiritual and weave spirituality into their teaching or practice [8]. Indeed, modern yoga is promoted to all cross-sections of society and adapted to suit all faiths and none. Practitioners are often also interested in world peace [9]. In 2004, the number of yoga practitioners in the UK was estimated at over 2.5 million with no more up-to-date figures available [10,11]. Worldwide, yoga as an industry is estimated to be worth US $ 80 billion [12].

This paper proceeds in the following order: first, the relation between yoga and animals is discussed; second, diet is considered in respect to animals and yoga; third the methodology is outlined; fourth, the results are reported; and fifth, the results are discussed and conclusions of the study are drawn.

## 2. Yoga and Animals

Traditional and some forms of modern yoga relate to animals in numerous ways; here, four key ways are stipulated. Firstly, the principle of *ahimsa* forms the first *yama* (ethical restraint) in *Patañjali’s Yoga Sūtra*, which has become the foremost text in modern yoga after its popularisation in the 19^th^ century [4,13]. According to Sanskrit norms, items listed first carry the most weight and inform what follows [14]. Thus, *ahimsa* can be considered as the most important *yama* and to inform all subsequent teachings. Moreover, other teachings such as mental discernment (*viveka)* and all subsequent *yamas* and *niyamas*, such as non-stealing (*asteya*) and non-greed (*aparigraha*), can be applied to human-animal relations as well as to between humans. 

Secondly, the etymological root of *yoga* is ‘yuj’, one meaning of which is to yoke or unite. Some yoga traditions relate this union to the aim of abiding purely as the spiritual Self [15]. Other yoga traditions relate it to an underlying Oneness of everything, which can promote the union between a perceived Infinite within each person to a perceived Infinite within all creatures [1]. All yoga philosophies accept a belief in the transmigration of the soul in yoga, whereby the soul continues living and is re-born into another body, which could frequently be that of another animal species [16]. Hindu scholars have also highlighted the importance of ethical relationship in yoga; that is, how practitioners relate to other sentient beings in an empathetic capacity [16].

Thirdly, an abundance of animal-named postures exist in yoga. There are over 70 recognised animal-named postures such as fish pose (*matsyasana*), camel pose (*ustrasana*) and crow pose (*kakasana*). Indeed, some of the oldest postures documented are animal-named postures, with the very first non-seated postures having animal names and dating back to c. 800 CE [1]. A key *hatha* yoga text, *The Gheranda Samhita*, states that the number of yoga postures equates to the number of species of living beings [17]. Moreover, the text *Patañjali’s Yoga Sūtra* (c. 400 CE) features three animal-named postures, and later commentaries on this text instruct learning the animal postures through the observation of animals [1]. There is also at least one animal-named breath practice, namely the bee breath (*bhramari pranayama)*, and numerous animal-named hand gestures such as the dove gesture (*kapota mudra*) [18,19].

Fourthly, vegetarianism is encouraged within yogic texts such as the 15^th^ century text *Hatha Yoga Pradipika* and by numerous leading western and non-western yoga leaders such as Sharon Gannon [14]. Vegetarianism is believed to enhance the *sattwic* (calm) quality that yoga seeks to optimise in practitioners [16]. Cows are thought to represent this sattwic quality, which is one reason why cows are afforded a high status within yogic Indic cultures [20]. India has the largest concentration of vegetarians in the world with up to 40% of its population abstaining from eating meat and fish, which may be related to economic factors as well as moral and faith-based considerations [21]. In 2016, the yoga scholar Dickstein made the case for vegetarianism based on *Patnñjali’s Yoga Sūtra*; he also suggested that, in modern times, a vegan diet could arguably be most aligned with *Patañjali’s Yoga Sūtra* [22]. In 2019, he strengthened his call for veganism within modern yoga [23]. 

Based on the aforementioned connections between yoga and animals, other yoga scholars also propose the inclusion of all non-human animals into humanity’s moral sphere and vegetarianism (increasingly veganism) as the most complementary lifestyle and outlook for a yogic path [16,24,25,26]. Animalia Asana^®^ (Fife, Scotland), Yogific (London, UK) and Yoga for Nature™ (Sydney, Australia) are examples of yoga charities, companies or groups promoting veganism based on these connections between yoga and animals. In contrast, however, Jarow [27] describes a counter-voice in yoga communities that accepts non-vegetarianism. This research concerns the beliefs and attitudes of UK yoga teachers regarding plant-based diets. The different dietary categories related to the consumption of animal protein shall now be introduced followed by a close examination of the reasons cited by yoga teachers, scholars and practitioners for and against a plant-based diet.

## 3. Dietary Categories Related to Abstention from Animal Products

Table 1 details descriptions of diets followed in the UK and other western countries. The Food Standards Agency has reported that 3% of the UK population identified as strict vegetarian and 1% as vegan in 2017 [28]. The Vegan Society [29] similarly estimated in 2018 that just over 1% (1.16%) of the UK population is vegan. It estimates that the number of vegans in the UK quadrupled between 2014 and 2018 [29]. Vegetarians abstain from consuming meat products and often fish and other marine life. Vegans do not use animal products of any kind as far as is practically possible. The British Dietetic Association [30] defines a plant-based diet as a diet ‘based on foods derived from plants, including vegetables, wholegrains, legumes, nuts, seeds and fruits, with few or no animal products’. Definitions for these and other dietary types are summarized in Table 1.

The number of yoga teachers in the UK is unknown but was estimated at around 10,000 in 2013 [31]. There is a significant gap in research on UK yoga teachers’ or practitioners’ dietary habits and attitudes towards plant-based diets. Ross et al., in 2013 [32] and Cramer et al. in 2017 [33] have surveyed yoga practitioners in the USA and Australia, respectively. Both studies found a higher prevalence of different forms of vegetarianism amongst yoga practitioners compared to their general populations. For example, 10% of US yoga practitioners were vegetarian, which is over three times the prevalence in the general US population [32]. In an Australian study from 2012, Penman et al., [9] found that yoga practitioners tended to be middle-class, highly-educated, health conscious, idealistic and interested in world peace and meditation.

## 4. Should Yoga Teachers and Practitioners Consume Animal Products? 

There is debate in the international yoga community about whether yoga teachers and practitioners should consume animal products or aim towards following a vegetarian or plant-based diet [14,22,26,34,35]. Some who believe yoga teachers should aim towards vegetarianism or veganism cite the inclusion of all other sentient beings in the application of *ahimsa*. They argue that yoga teachers are in a position of responsibility and influence and should act in accordance with these beliefs. They also argue for seeing ourselves in other animals and point to the yogic teaching of reducing suffering [14,36]. It is also argued that a healthy vegan diet is necessary for the ultimate goal of liberation [22]. In 2019, yoga scholar Dickstein emphasised the need for yoga communities to ‘see’ the individual animal victims before they become so-called food [23]. He acknowledged other progressive and conscious segments within the modern yoga community, such as abuse-awareness and ‘decolonising’ yoga initiatives, and expressed surprise and disappointment that the yoga community does not embrace veganism more in its sense of social justice. Globally, both yoga practice and plant-based diets are experiencing upward trends in the number of people adopting them [37]. 

Factors related to health or illness are cited by some yoga teachers championing the ethics of eating animal products [38]. Proponents also appear to apply *ahimsa* chiefly towards themselves and accuse those who believe yoga teachers should be vegetarian as judgmental, which they claim is un-yogic [35,39]. Tsung [38] claims that there is even a kind of taboo regarding eating meat as a yoga teacher. Moreover, a need to accept violence in the world and all forms of food as equally pure is emphasised [27]. In 2019, Dickstein referred to a growing tendency within the modern yoga community to conflate promotions of veganism or plant-based diets with dogma to which there is a strong aversion [23]. 

In a 2018 USA survey-based study, Cramer et al., [40] examine the differences between omnivorous and vegetarian yoga practitioners. They found that a higher proportion of vegetarian yoga practitioners (72.2%) compared to non-vegetarian yoga practitioners (53.4%) included meditation as part of their yoga practice. They also found that more vegetarians ate organic food (46.8% vs. 22.2%), used yoga to feel better emotionally (80.3% vs. 65.2%), used yoga because it was natural (80.9% vs. 60.9%) and incorporated breathing exercises into their practice (91.8% vs. 89.5%). They suggest that vegetarian yoga practitioners may be more likely than their omnivorous counterparts to view yoga as a lifestyle than as therapy alone and to practice the other ‘limbs’ of yoga, not just the postures.

## 5. Materials and Methods 

### 5.1. Research Questions and Design 

This research aims to investigate the dietary habits, beliefs about the moral status of farmed animals and attitudes towards plant-based diets of UK yoga teachers. Yoga teachers rather than yoga practitioners were chosen as the subjects for this study because they (i) will most likely have a more nuanced understanding and developed practice of yoga; (ii) play a role in shaping how yoga evolves; and (iii) have the potential to positively influence yoga practitioners. The research questions are as follows:

(1). What are UK yoga teachers’ beliefs about the moral status of farmed animals? 

(2). What are UK yoga teachers’ dietary habits and attitudes towards plant-based diets?

(3). What is the relation between UK yoga teachers’ beliefs about the moral status of animals, their dietary habits and their attitudes towards plant-based diets? 

(4). What is the relation between UK yoga teachers’ knowledge of animal agriculture, their dietary habits and their attitudes towards plant-based diets?

(5). What are the barriers for UK yoga teachers to transitioning to a more plant-based diet?

A sequential mixed-methods approach was used to investigate these research questions. Phase 1 of the research employed a questionnaire to investigate the research questions using a quantitative methodology. Phase 2 used semi-structured interviews to provide a deeper analysis using a qualitative approach. This paper reports the results of Phase 1 based on the questionnaire methodology. The first author is a plant-based yoga teacher and lecturer in animal welfare science, ethics and law. The second author is an academic specialising in human animal studies.

### 5.2. Questionnaire

A questionnaire comprising 18 closed questions and one open question was used in this research. Henceforth, ‘Q’ refers to the question number(s) in the questionnaire. Q1–9 concerned demographic data and information about yoga teaching and practice from the respondents. Table 2 summarises Q10–17. Brief definitions were provided for the nine dietary types in Q10. The definition of a plant-based diet was ‘I avoid consuming all animal products and aim for a 100% plant-based diet’. The vegan definition was ‘I do not eat animal products at all’. The points on each Likert scale or series of Likert-type items were labelled with a description of what they represented (e.g., ‘strongly agree’). Q18–19 asked for the respondents’ contact details and if they either were willing to be interviewed for the next phase of the research or would like to be informed of the results of the research. 

SurveyMonkey was used to create and house the survey, and it was open to respondents from March to May 2018. The questionnaire was piloted on three individuals and then posted on the Yoga Teachers UK Facebook group, which at the time of the research had over 6000 members. A respondent was eligible for participation in this research if they resided in the UK and had completed a yoga teacher training qualification. Ethical approval for conducting the research was received from the University of Winchester Research Ethics Committee.

The population of yoga teachers in the UK is unknown but was estimated to be around 10,000 in 2013 [31]. The first author also arrived at a figure of around 10,000 UK yoga teachers by extrapolating from the USA yoga teacher population; this involved applying the percentage of the total USA population that classifies themselves as yoga teachers to the UK population [41]. To optimise external validity, an online sample size calculator was used to arrive at a target sample size, so the results could be generalised. A 5% margin of error and a 95% confidence level were selected. The resulting target number of participants for the survey was 370 assuming a 20% response rate from 1850 invites. 

### 5.3. Data Analysis

The data were exported into IBM SPSS Statistics 25. The data of any respondent who missed out all questions after the first four were deleted. Four larger dietary groups were formed from the nine dietary categories in the questionnaire as follows: (i) Omnivore (Traditional UK diet; Conscientious omnivore; Reducetarian; Pollotarian; Pescatarian; Avoid dairy and eggs but eat meat, fish and seafood), (ii) Vegetarian (Vegetarian), (iii) Plant-based (Vegan; Plant-based) and (iv) Other (Other). 

Respondents following plant-based and vegan diets were grouped together as they were deemed sufficiently similar in terms of purchasing and consumption habits. Plant-based was chosen for the title of this dietary group as this paper is focused on diet as opposed to other aspects of a vegan lifestyle. All categories subsumed under the constructed omnivore group are also similar in that they involve the consumption of animal protein from killed animals. The vegetarian group included the single dietary category of ‘Vegetarian’ in the questionnaire. Due to the mixed constitution of the ‘Other’ category, this was not merged with another category.

#### 5.3.1. The Animal Belief Scale

Q13 was used to answer the first and second research questions by grouping the 26 items into their respective categories: (i) beliefs about the moral status of farmed animals and (ii) attitudes towards plant-based diets. At this point, two items (concerning beliefs about lab meat and entomophagy) were deemed insufficiently relevant to the topics of concern and so were left out of all analyses. Two 12-item Likert scales were then formed from the aforementioned groups of items. The first scale measures the extent of progressive beliefs regarding the moral status of farmed animals. For the purposes of this research, it is named the Animal Belief Scale. On this scale, the higher the score, the more progressive a belief about the moral status of farmed animals. In this research, the more aligned a belief is to the reduction of the suffering and killing of sentient non-humans, the more progressive it is deemed to be.

#### 5.3.2. The Plant-Based Diet Scale

The second scale measures the extent of positive attitudes towards plant-based diets. For the purposes of this research, it is named the Plant-Based Diet Scale. On this scale, the higher the score, the more positive an attitude towards plant-based diets is deemed to be. The language orientation of half of the items on both the Animal Belief Scale and the Plant-Based Diet Scale was converted to positive to ensure all statements measure in the same direction. The answer codes of all respondents for these altered statements were then reverse coded to match the intended rating of the yoga teachers.

To investigate the third research question, the Pearson’s chi-squared test was used to determine any significant relationship between each of the belief and attitude items in Q13 and diet group. If an assumption of this test was violated (over 20% of the cell expecteds were less than five), the likelihood ratio chi-squared test was used. Cramer’s V was used to determine the strength of any relationships found. 

Three median scores per respondent for each of the three scales in this study were computed as new variables and then used in non-parametric tests. A mean value for each scale was also determined from these median values. The Kruskal-Wallis test was performed to test for significant differences amongst the diet groups in their median scores on the Animal Belief Scale and Plant-Based Diet Scale. The Dunn-Bonferroni post-hoc test was performed to reveal which dietary groups the statistically significant differences were between. The Spearman’s rho test was used to test for correlations between the participants’ beliefs about the moral status of farmed animals and their attitudes towards plant-based diets.

#### 5.3.3. The Agricultural Knowledge Scale

To investigate the fourth research question, the items of Q15 were also grouped to form the Agricultural Knowledge Scale. The higher the score on the Agricultural Knowledge Scale, the higher the level of awareness a respondent was deemed to have regarding different aspects of animal and plant agriculture. Pearson’s chi-squared test (or the likelihood ratio chi-squared test where applicable) and Cramer’s V were also used to determine any relationships and the strength of any relationships between individual agricultural knowledge items and diet group. The Kruskal-Wallis test was then performed to test for statistically significant differences amongst the diet groups in their median scores on the Agricultural Knowledge Scale. The Dunn-Bonferroni post-hoc test was used to reveal which diet groups the significant differences were between. To investigate the fifth research question, the responses to the open-ended Q17 were quantified by tallying their frequency of occurrence. Key themes in the responses were then identified.

## 6. Results

After data cleaning, 449 UK yoga teachers responded to the questionnaire. Barring questions 11–12, which were only for those following a plant-based or vegan diet, the number of respondents for each question ranged from 385–449. Over 35% of yoga teachers were aged between 35 and 44 (n = 156). This formed the largest age group of yoga teachers, followed by ages 45–54 (32%, n = 141). Over 88% (n = 393) of yoga teachers were female and less than 10% were male (n = 41). Over 76% of yoga teachers had completed university-level education with 39.4% (n = 175) selecting an undergraduate degree and 36.7% (n = 163) selecting a postgraduate degree as their highest education level. This demographic information is representative of the international yoga teaching and practitioner population, which has a significant female-oriented gender imbalance outside of India and comprises predominantly middle-aged well-educated individuals [37]. 

### 6.1. UK Yoga Teachers’ Beliefs about the Moral Status of Farmed Animals

Table 3 illustrates participants’ responses to the statements in Q13 concerning the moral status of farmed animals. For example, item 7 states that ‘Minimising animal suffering is just as important as minimising human suffering’. In response, 86% of participants agree (64.8% completely agree; 21.2% somewhat agree). Item 10 states that ‘Killing an animal in a sacred way can enhance one’s spirituality’. In response, 88.4% of participants disagree (78.4% completely disagree; 10.0% somewhat disagree). See Section 6.3 for statistical analysis using the chi-squared test and Cramer’s V represented in Table 3.

#### Animal Belief Scale

A high Cronbach’s alpha of 0.756 suggests internal consistency between the items in Table 3. These items were therefore grouped to form the Animal Belief Scale (item two from Table 3 was excluded from the Animal Belief Scale due to the statement comprising both progressive and conservative elements). In the Animal Belief Scale, ‘completely disagree’ correlates with a value of 1 and ‘completely agree’ correlates with a value of 5 for a statement that indicates progressive beliefs. The Animal Belief Scale mean value is 4.64. This correlates with UK yoga teachers on average varying between ‘somewhat agreeing’ and ‘completely agreeing’ with progressive statements about the moral status of animals.

### 6.2. UK Yoga Teachers’ Dietary Habits and Attitudes Towards Plant-Based Diets

**Figure 1:**
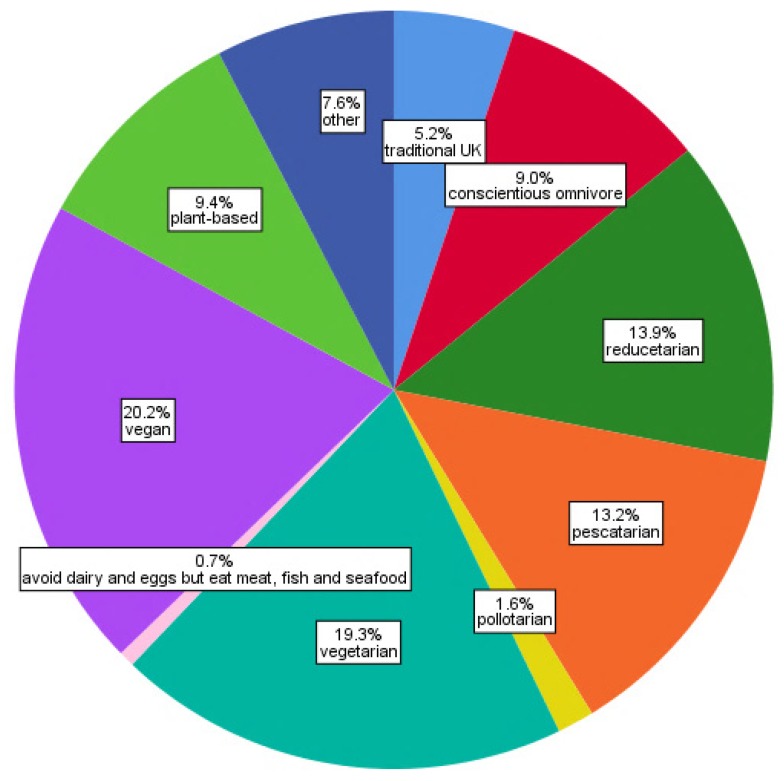
Pie chart illustrating UK yoga teachers’ dietary habits (n = 446).

**Figure 2:**
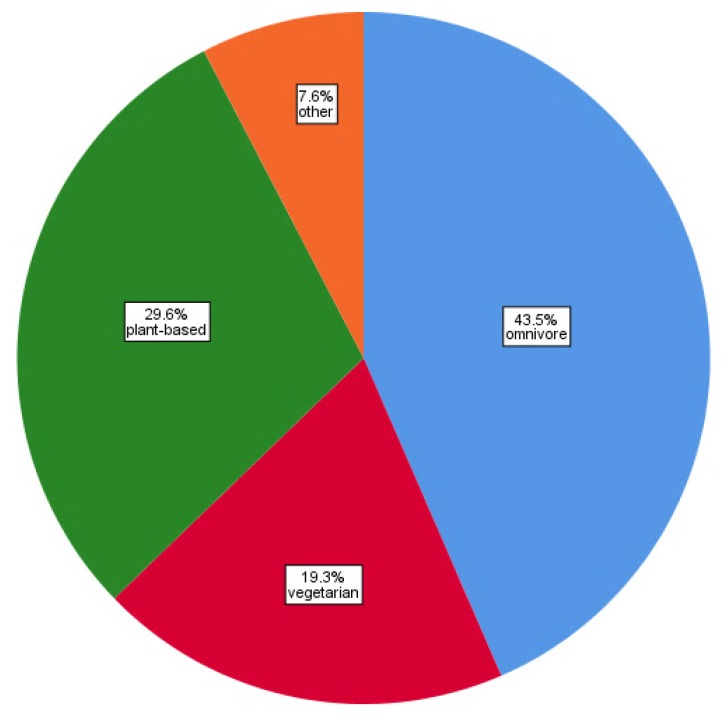
Pie chart illustrating UK yoga teachers’ grouped dietary habits (n = 446).

Figure 1 illustrates the dietary patterns of UK yoga teachers based on responses to Q10. The two largest dietary categories are vegan (20.2%) and vegetarian (19.3%). The smallest dietary category of respondents is avoid dairy and eggs but consume meat, fish and seafood (0.7%). In Figure 2, the dietary categories have been formed into the following groups: (i) Omnivore, (ii) Vegetarian, (iii) Plant-based and (iv) Other (also see Methodology). The largest combined group is omnivores (43.5%), followed by plant-based (29.6%) and then vegetarian (19.3%). These grouped dietary categories will be discussed in the remainder of this paper.

Table 4 illustrates participants’ responses to the statements in Q13 concerning attitudes to plant-based diets. For example, item 1 states that ‘I would like to follow or continue to follow a plant-based or vegan diet’. In response, 73.9% of participants agree (49.5% completely agree; 24.4% somewhat agree). Item 6 states that ‘A plant-based diet is most aligned with my yoga practice’. In response, 68.6% agree (40.7% completely agree; 27.9% somewhat agree). See Section 6.3 for statistical analysis using the chi-squared test and Cramer’s V represented in Table 4.

#### Plant-Based Diet Scale

A high Cronbach’s alpha of 0.743 suggests internal consistency between the attitude items in Table 4. These items were thus grouped to form the Plant-Based Diet Scale. The mean Plant-Based Diet Scale value is 4.25. This correlates with UK yoga teachers on average varying between ‘somewhat agreeing’ and ‘completely agreeing’ with statements of positive attitudes to plant-based diets.

### 6.3. UK Yoga Teachers’ Beliefs about the Moral Status of Animals, Dietary Habits and Attitudes Towards Plant-Based Diets

Table 3 illustrates how each chi-square statistic for each belief item is greater than 21.03. Hence, the chi-squared tests reveal a significant relationship between each belief about the moral status of farmed animals and diet group. Cramer’s V finds that this relationship is strong (>0.3) for two items (2 and 11), moderate (0.2–0.3) for seven items (1, 3–6, 9 and 12) and weak (<0.2) for three items (7–8 and 10). 

Table 4 illustrates how each chi-square statistic for two thirds of the items (8 out of 12) is higher than 21.03. Hence, the chi-squared tests reveal a significant relationship between attitude items 1–2 and 5–10 and diet group. Cramer’s V finds that the relationship is strong (>0.3) for items 1–2 and 6–7, moderate (0.2–0.3) for items 5, 8 and 10 and weak (<0.2) for item 9. The Kruskal-Wallis test indicates that the respondents’ median scores of each scale are significantly different across diet group (H = 96.409 on the Animal Belief Scale; H = 149.657 on the Plant-Based Diet Scale; 3 df, *p* = 0.00). On the Animal Belief Scale, the Dunn-Bonferroni post-hoc test indicates that the plant-based group is the highest ranking and the omnivore group is the lowest ranking. It also reveals significant differences between the omnivore and vegetarian groups, and between the omnivore and plant-based groups (see Table 5). There are no significant differences between the vegetarian and plant-based groups.

On the Plant-Based Diet Scale, the Dunn-Bonferroni post-hoc test indicates that the plant-based group is the highest ranking grouped dietary category and the omnivore group is the lowest ranking group. It also found significant differences between omnivore and vegetarian groups, omnivore and plant-based groups, and vegetarian and plant-based groups (see Table 6). A positive correlation between the respondents’ median values for the Animal Belief Scale and the Plant-Based Diet Scale was also found (Spearman’s rho = 0.431, *p* = 0.000).

### 6.4. The Relation Between UK Yoga Teachers’ Knowledge of Animal Agriculture, their Dietary Habits and their Attitudes to Plant-Based Diets

Table 7 illustrates participants’ responses to statements in Q15 concerning knowledge of agriculture. For example, item 1 states that ‘Global animal agriculture is considered responsible for at least 14% of global greenhouse gas emissions; this is more than the whole of the transport sector’. In response, 76% of participants are aware (50.4% fully aware; 25.6% somewhat aware).

Table 7 also shows that the chi-square statistic for each item is higher than 21.03. Hence, the chi-squared tests reveal a significant relationship between each item of self-reported knowledge about agriculture and diet group. Cramer’s V finds that this relationship is moderate (0.2–0.3) for items 3–5 and 8–9 and weak (<0.2) for items 1–2, 6–7, and 10–11.

#### Agricultural Knowledge Scale

A high Cronbach’s alpha of 0.898 suggests internal consistency between the items in Table 7. Therefore, they were grouped to form the Agricultural Knowledge Scale. The Agricultural Knowledge Scale mean value is 4.09. This value correlates with UK yoga teachers on average varying between being ‘somewhat aware’ and ‘fully aware’ of practices in animal and plant agriculture. Significant differences were suggested by the Kruskal-Wallis test between yoga teachers of different diet groups regarding their median scores on the Agricultural Knowledge Scale (H = 73.484, 3 df, *p* = 0.00). The Dunn-Bonferroni post-hoc test reveals that agricultural knowledge ranked highest amongst plant-based yoga teachers and lowest amongst omnivore yoga teachers. It also reveals significant differences between omnivores and vegetarians, between omnivores and plant-based individuals and between vegetarians and plant-based individuals (see Table 8.).

### 6.5. Barriers to Transitioning to a Plant-Based Diet

**Figure 3:**
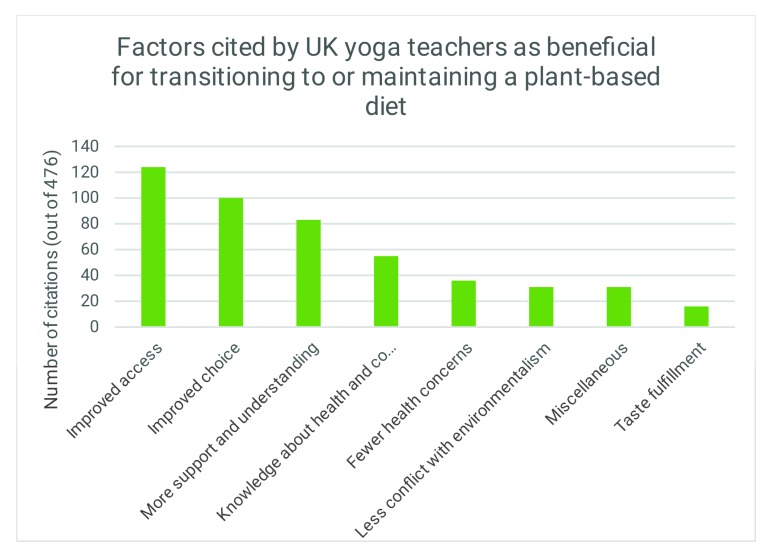
Bar chart illustrating the factors cited by UK yoga teachers as beneficial for transitioning to or maintaining a plant-based diet.

The participants cited 476 reasons in total that could help with transitioning to or maintaining a plant-based diet. The frequency of these reasons were tallied and grouped into the following themes: 1) improved access to plant-based foods (26%, n = 124), 2) improved choice of plant-based foods (21%, n = 100), 3) more support and understanding from others (17%, n = 83), 4) more knowledge regarding health and cooking (12%, n = 55), 5) fewer health concerns (8%, n = 36), 6) less perceived conflict with environmentalism (7%, n = 31), 7) miscellaneous (7%, n = 31) and 8) taste (3%, n = 16). Thematic reasons provided by UK yoga teachers to help transition to or maintain a plant-based diet are illustrated in Figure 3.

## 7. Discussion

### 7.1. Beliefs about the Moral Status of Farmed Animals

This research has found that UK yoga teachers have very progressive beliefs about the moral status of farmed animals. For instance, 86% agree that ‘Minimising animal suffering is just as important as minimising human suffering’. Furthermore, over 68% of UK yoga teachers agree that a plant-based diet is most aligned with their yoga practice. Together, these results suggest that a majority of UK yoga teachers interpret yogic teachings such as *ahimsa* in a way that places the suffering of non-human animals on an equal footing with that of humans. Peter Singer has argued that we should apply equal consideration to the interests of sentient non-human animals [42]. He argues that considering the suffering of humans as more morally important than the equivalent suffering of non-human animals is speciesist. The research finds that UK yoga teachers have beliefs consistent with Singer in this respect. Further research is required to be more confident in the precise reasons why UK yoga teachers find a plant-based diet as most congruent with their yoga practice. 

These fundamental moral beliefs relating to the equal moral standing of human and non-human animals also relate to more practical attitudes towards farmed animals. For instance, 78.6% of respondents agree that ‘Farmed animals in the UK do not have good lives’. Furthermore, 92.9% of respondents agree with the statement that ‘The intensive farming of animals is morally wrong’. These findings can be compared with the research commissioned by the European Commission [43], which found that 76% of the UK general public think the welfare of farmed animals in the UK should be better protected than it is now.

UK yoga teachers also have progressive views on the killing of non-human animals. Over half (52.8%) agree that ‘If plant-based foods are available, it is impossible to kill an animal humanely’. Furthermore, 78.2% agree that ‘It is not more morally acceptable to eat some animals than others’. This finding contrasts UK yoga teachers with the wider population, who prioritise the interests of some species over others. For instance, on a global basis society tends to abstain from consuming dogs and cats [44]. Similarly, great apes, elephants and cetaceans are generally considered to have a higher moral status than species commonly farmed for food [45].

Despite the emphasis on *ahimsa* and unity between all beings, many Hindus in particular have practiced and some continue to practice animal sacrifices to the gods; for example, as part of the Gadhimai Festival in Nepal or Durga Puja celebrations in eastern India. Indeed, Buddhism and Jainism are both antithetical to Hinduism in this regard in that they are opposed to the practice of animal sacrifice [26]. Item 10 of Table 3 reads ‘Killing an animal in a sacred way can enhance one’s spirituality’. A large majority (88.4%) of respondents disagree with this statement, with 78.4% strongly disagreeing and 10.0% somewhat disagreeing. Despite yoga’s associations with Hinduism, this finding suggests that modern yoga teachers in the UK have not adopted pro animal sacrifice beliefs. Instead, more akin to Jains and Buddhists, modern yoga teachers in the UK reject the necessity and validity of killing an animal for the enhancement of a spiritual purpose.

Some faiths and cultures such as some branches of tantric Buddhism believe that through the consumption of animal flesh, humans can practice accepting and surrendering to the inevitable coil of *samsara* (suffering) and *himsa* (violence) and non-attachment to purity [27]. Consequently, humans embrace and connect to human animal nature and the nature of the world to the fullest [27]. However, in this research, in response to the statement ‘Eating animals connects us with nature’, 85.1% of respondents disagree (75.3% completely disagree; 9.8% somewhat disagree). Hence, a substantial majority of surveyed UK yoga teachers do not believe that eating animals connects us to nature.

### 7.2. Dietary Habits and Attitudes Towards Plant-Based Diets

This research found that 43.5% of UK yoga teachers surveyed are omnivores, 29.6% consume a plant-based diet and 19.3% are vegetarians. In the wider UK population, 3% are vegetarian and just over 1% (1.16%) are vegan, meaning around 96% are omnivores. Hence, our research found a far greater proportion of UK yoga teachers who abstain from consuming animal products compared to the wider UK population. Indeed, the proportion of UK yoga teachers that are vegetarian is six-fold higher than in the UK general public, whilst the proportion of UK yoga teachers that are vegan or plant-based is 25-fold greater than in the general public. Furthermore, in the UK population, there is more than double the number of vegetarians (3%) than vegans (around 1%). In contrast, this research finds there are substantially more (around 50% more) UK yoga teachers following a plant-based diet compared to vegetarians.

The results therefore reveal not only a far higher proportion of UK yoga teachers following a plant-based or vegetarian diet, but also a substantially higher proportion following a plant-based/vegan diet compared to vegetarians. The latter is an interesting finding because it is contrast to the situation in the wider UK public. Most vegans abstain from the consumption of animal products for moral reasons relating to concern about the killing or suffering of non-human farmed animals [46,47]. Ethical vegans tend to follow an animal rights philosophy [48], which holds that sentient non-humans have a moral right to not suffer and not be killed [49,50]. Vegetarians, who normally consume eggs and dairy products, hold similar concerns about killing animals and their suffering [51]. However, the beliefs that vegetarians and vegans hold may differ both in kind and in the strength of conviction [48]. This research suggests that the far higher proportions of UK yoga teachers following a plant-based or vegetarian diet relative to the wider UK public relates to yogic teachings being applied to dietary choices. The most relevant and obvious teaching in this regard is the principle of *ahimsa* or non-harm to all beings. Thus, UK yoga teachers may specifically be applying the principle of *ahimsa* to their dietary choices to refrain from causing harm in a number of ways to sentient farmed animals. More research is required to be more confident that it is the specific teaching of *ahimsa* influencing this behaviour rather than other yogic teachings.

### 7.3. Beliefs about the Moral Status of Animals, Dietary Habits and Attitudes Towards Plant-Based Diets

This research found statistically significant relationships between questionnaire items about the moral status of farmed animals and diet group. An example of one belief item that was found to have a strong relationship to grouped dietary category is ‘If plant-based foods are available, it is impossible to kill an animal humanely’. Similarly, this research revealed significant relationships between individual items about attitudes towards plant-based diets and diet group. For example, the statement ‘A plant-based diet is most aligned with my yoga practice’ was found to have a strong relationship to grouped dietary category. 

The research also found that UK yoga teachers’ median scores on the Animal Belief Scale and Plant-Based Diet Scale were significantly different between the various diet groups. On both composite scales, plant-based yoga teachers ranked the highest and omnivores the lowest. For both scales, there were significant differences between omnivores and vegetarians, and between omnivores and plant-based individuals. Additionally, on the Plant-Based Diet Scale, there were significant differences between UK yoga teachers following vegetarian and plant-based diets. 

Together, these results indicate that UK yoga teachers with more progressive views about the moral status of farmed animals and more positive attitudes towards plant-based diets abstain from consuming a greater range of animal products in their diets. This is consistent with previous studies that have revealed differences in beliefs about the moral status of animals between different dietary groups amongst the UK general public [48].

### 7.4. Knowledge about Agriculture, Dietary Habits and Attitudes to Plant-Based Diets

The research finds that UK yoga teachers have a high level of self-reported knowledge about animal and plant agriculture. For example, item 3 of Table 7 reads ‘In the UK, thousands of male dairy calves are shot, and millions of male chicks are gassed annually, because they are worthless to the dairy and egg industries’. In response to this, 71.5% of respondents were aware (54.9% fully aware; 16.6% somewhat aware). The Agricultural Knowledge Scale further reinforces this. In this scale, a value of 1 represents the lowest level of knowledge about agriculture and 5 represents the highest level. Given that the Agricultural Knowledge Scale mean value is 4.09, it suggests that UK yoga teachers are somewhat aware to fully aware of animal and plant agricultural practices. 

The research also suggests a significant relationship between each item of self-reported knowledge about animal and plant agriculture and diet group. It found that UK yoga teachers following a plant-based diet had the highest levels of agricultural knowledge, and omnivorous yoga teachers had the lowest levels of agricultural knowledge. This suggests that the higher the level of knowledge yoga teachers have regarding animal and plant agriculture, the greater extent to which they are likely to have eliminated animal products from their diet. Both modern and traditional yoga promote qualities of truth, knowledge, awareness, discernment, study and responsibility [22,52]. Thus, these results may be explained by yoga teachers’ efforts to incorporate these qualities into their lives.

## 8. Conclusions

Yoga is a physical, mental and spiritual discipline that originated in ancient India. It is chiefly associated with Hinduism, but is also a component of other faiths and cultures, most notably Buddhism and Jainism. Modern yoga as practiced in the UK and western world primarily focuses on health and relaxation. Both traditional and modern forms of yoga share a belief in *ahimsa*, meaning non-harm to all beings. This has led to debate in the international yoga community about whether the principle of *ahimsa* prescribes that teachers and practitioners should abstain from consuming animal products and consume a plant-based diet.

This questionnaire-based research surveyed the beliefs and attitudes of yoga teachers in the UK about the moral status of farmed animals, attitudes to plant-based diets and knowledge about agriculture. UK yoga teachers have very progressive beliefs about the moral status of farmed animals and positive attitudes towards plant-based diets. For instance, 86% of UK yoga teachers surveyed agree that minimising animal suffering is as important as minimising human suffering. Furthermore, over 68% of UK yoga teachers agree that a plant-based diet is most aligned with their yoga practice.

UK yoga teachers with more progressive beliefs about the moral status of farmed animals abstained from consuming animal products to a greater extent than those with less progressive views. Similarly, there was a positive correlation between self-reported knowledge about agriculture and abstention from consuming animal products. 

In the wider UK population, 3% of the public are vegetarian and 1% follows a vegan diet. For UK yoga teachers surveyed, 19.3% identify as vegetarian and 29.6% follow a plant-based diet. Thus, the proportion of vegetarians in the UK yoga teaching populations is six-fold higher compared to the wider UK population. The proportion of UK yoga teachers following a plant-based diet is 25-fold higher than the general UK population. Additionally, whilst there are three times more vegetarians than vegans in the wider UK population, for UK yoga teachers there are one and a half times the number of people following a plant-based or vegan diet compared to vegetarians. 

The far higher proportions in the UK yoga teaching community of both vegetarians and those following a plant-based diet, relative to the wider population, are likely based on yogic teachings such as the principle of *ahimsa*, meaning non-harm to all beings.

## Figures and Tables

**Table 1:** Dietary classifications related to the consumption of animal products used in the UK, Europe and the USA.

Diet	Description
Standard omnivore	Consumes many types of animal product according to cultural norms
Conscientious omnivore	Consumes many animal products according to cultural norms but aims to procure from higher welfare and local sources
Reducetarian	Aims to reduce the consumption of animal products such as meat, dairy and fish
Pescatarian	Restricts consumption of animal products to fish, other marine life, eggs and dairy products
Pollotarian	Restricts consumption of animal products to poultry, fish, other marine life, eggs and dairy products
Vegetarian/lacto-ovo-vegetarian	Restricts consumption of animal products to eggs and dairy products
Vegan	Abstains from all consumption and use of animal products
Plant-based	Consumes foods derived from plants, including vegetables, wholegrains, legumes, nuts, seeds and fruits, with few or no animal products

**Table 2:** Questionnaire instrument including items, question wording and measurement scales.

Item(s) and Question Number	Question Wording	Measurement Scale
Diet type (Q10)	Which option do you think best describes your general diet?	10 categories: Standard UK diet; Conscientious omnivore; Reducetarian; Pescatarian; Pollotarian; Vegetarian; I avoid consuming dairy and eggs, but I eat meat, fish and/or seafood; Vegan; Plant-based; Other (please state)
Important dietary transition factors (Q11)	Please rank how important the following factors were in aiding your transition	A series of eight 5-point Likert-type items: 1 = the least important, 5 = the most important (e.g., knowing other vegans, having support from family and planning meals in advance)
Length of time following a plant-based or vegan diet (Q12)	Roughly how long have you been following a plant-based or vegan diet?	Five categories: <6 months, 6 months to 1 year, 2 to 4 years, 5 to 7 years or Over 7 years
Beliefs about the moral status of farmed animals and attitudes towards plant-based diets (Q13)	Please tick the box that most closely reflects how much you agree or disagree with the following statements	A 26-item 5-point Likert-type scale from strongly disagree to completely agree (e.g., ‘Farm animals in the UK have good lives’)
Important factors in everyday food choices (Q14)	Please select the three factors from the table below that you consider to be the most important when making your everyday food choices?	Nine options: Taste; Cost; Health; Having animal products in every meal; Minimising animal products; Eco-friendliness; What family/friends are eating; Convenience; Familiarity
Awareness of agricultural practices (Q15)	Please state how aware of the following true statements you were prior to completing this questionnaire	An 11-item 5-point Likert scale: ‘1’ indicates not at all aware; ‘5’ indicates fully aware (e.g., ‘Global animal agriculture requires three times as much land and water as plant-based diets do’)
Changing attitudes towards plant-based diets (Q16)	Do you think your attitudes towards plant-based diets and food habits may alter upon further reflection of the information shared in the previous question?	Four options: Yes, No, Maybe or Other (please state)
What could help the transition to a plant-based diet? (Q17)	Please finish this sentence with your thoughts	For me to transition to, or maintain, a more plant-based diet, it would be helpful if…

**Table 3:** UK yoga teachers’ beliefs about the moral status of farmed animals and relationship between belief items and diet group.

ItemNo.	Statement	Completely Disagree	Somewhat Disagree	Not Sure	Somewhat Agree	Completely Agree	Chi-sq. Test	Cramer’s V
1	We should consume animal products to protect human culture and tradition	69.9%	18.0%	6.4%	3.3%	2.3%	61.451 ***	0.225 ***
2	I support measures to increase animal welfare but not to reduce the consumption of animal products	35.8%	30.4%	10.3%	18.0%	5.4%	105.455 ***	0.301 ***
3	We should consume animal products to support livestock farmers	59.5%	22.7%	9.5%	7.5%	0.8%	96.175 ***	0.275 ***
4	Farm animals in the UK have good lives	40.2%	38.4%	12.1%	8.2%	1.0%	97.668 ***	0.285 ***
5	It is more morally acceptable to eat some animals than others	63.5%	14.7%	9.5%	8.5%	3.9%	98.136 ***	0.276 ***
6	Consuming milk and eggs is more morally acceptable than consuming meat	26.9%	24.6%	11.8%	30.2%	6.6%	83.442 ***	0.267 ***
7	Minimising animal suffering is just as important as minimising human suffering	2.6%	6.6%	4.8%	21.2%	64.8%	36.401 ***	0.175 ***
8	The intensive farming of animals is morally wrong	4.1%	1.8%	1.3%	11.0%	81.9%	48.821 ***	0.195 ***
9	It is impossible to safeguard farm animal welfare at the current level of demand for animal products	14.6%	12.3%	20.5%	23.5%	29.2%	55.712 ***	0.218 ***
10	Killing an animal in a sacred way can enhance one’s spirituality	78.4%	10.0%	7.7%	2.1%	1.8%	27.690 **	0.151 **
11	If plant-based foods are available, it is impossible to kill an animal humanely	12.1%	16.2%	18.8%	17.0%	35.8%	126.014 ***	0.329 ***
12	Eating animals connects us with nature	75.3%	9.8%	9.0%	5.1%	0.8%	76.373 ***	0.244 ***

Notes: 1. The likelihood ratio chi-squared test was used for items 1, 3–5, 7, 8, 10, 12; Pearson’s chi-squared test was used for the other items. 2. Levels of significance: * *p* < 0.05, ** *p* < 0.01, *** *p* < 0.001.

**Table 4:** UK yoga teachers’ attitudes towards plant-based diets and relationship between attitude items and diet group.

Item No.	Statement	Completely Disagree	Somewhat Disagree	Not Sure	Somewhat Agree	Completely Agree	Chi-sq. Test	Cramer’s V
1	I would like to follow or continue to follow a plant-based or vegan diet	7.2%	11.5%	7.4%	24.4%	49.5%	195.280 ***	0.409 ***
2	Following a plant-based diet is one of the most ethical choices we can make	3.8%	10.8%	7.9%	30.8%	46.7%	122.375 ***	0.308 ***
3	I know several people who follow a plant-based diet	1.8%	4.4%	1.3%	19.0%	73.6%	16.028	-
4	I like many different types of fruit and vegetable	1.0%	0.5%	0.5%	4.9%	93.1%	13.330	-
5	Plant-based diets are affordable	2.1%	13.1%	10.0%	25.7%	49.1%	69.735 ***	0.233 ***
6	A plant-based diet is most aligned with my yoga practice	7.2%	14.8%	9.5%	27.9%	40.7%	152.936 ***	0.361 ***
7	A small quantity of animal-derived foods is required for optimal health	39.1%	17.6%	13.6%	21.5%	8.2%	159.129 ***	0.368 ***
8	Governments should facilitate and normalise plant-based diets	4.4%	5.1%	12.6%	25.1%	52.8%	90.255 ***	0.265 ***
9	My personal dietary choice will not make a difference to farm animal welfare	36.8%	35.0%	10.2%	14.1%	3.8%	45.793 ***	0.180 ***
10	It is possible for everyone to consume a plant-based diet	9.0%	21.5%	15.1%	24.6%	29.9%	96.579 ***	0.287 ***
11	Singling myself out from my peers, friends and family is an obstacle to following a plant-based or vegan diet	42.8%	19.5%	9.2%	23.8%	4.6%	6.426	--
12	The convenience of accessing animal-derived foods is a barrier to following a plant-based or vegan diet	28.5%	22.1%	10.0%	28.5%	10.8%	15.686	-

Notes: 1. The likelihood ratio chi-squared test was used for items 2–5 and 8–9; the Pearson chi-squared test was used for the other items. 2. Levels of significance: * *p* < 0.05, ** *p* < 0.01, *** *p* < 0.001.

**Table 5:** Dunn-Bonferroni post-hoc test on median values of the Animal Belief Scale for UK yoga teachers in different diet groups.

Diet Type	Omnivore	Vegetarian	Plant-Based
Omnivore	-	Stat value -86.465 ** SE 14.431	Stat value -122.017 ** SE 12.389
Vegetarian	Stat value -86.465 ** SE 14.431	-	Stat value -35.552 SE 15.106
Plant-based	Stat value -122.017 ** SE 12.389	Stat value -35.552 SE 15.106	-

Note: Levels of significance: * *p* < 0.05, ** *p* < 0.01, *** *p* < 0.001.

**Table 6:** Dunn-Bonferroni post-hoc test on median values of the Plant-Based Diet Scale for UK yoga teachers in different diet groups.

Diet Type	Omnivore	Vegetarian	Plant-Based
Omnivore	-	Stat value -76.162 ** SE 15.069	Stat value -158.100 ** SE 12.937
Vegetarian	Stat value -76.162 ** SE 15.069	-	Stat value -81.938 ** SE 15.774
Plant-based	Stat value -158.100 ** SE 12.937	Stat value -81.938 ** SE 15.774	-

Note: Levels of significance: * *p* < 0.05, ** *p* < 0.01, *** *p* < 0.001.

**Table 7:** UK yoga teachers’ self-reported knowledge about animal and plant agriculture and the relationship between knowledge items and diet group.

ItemNo.	Statement	Not at All Aware	Not Very Aware	Neutral	Somewhat Aware	Fully Aware	Chi-sq. Test	Cramer’s V
1	Global animal agriculture is considered responsible for at least 14% of global greenhouse gas emissions; this is more than the whole of the transport sector	8.8%	4.1%	11.1%	25.6%	50.4%	42.427 ***	0.191 ***
2	Global animal agriculture requires three times as much land and water as plant-based diets do	6.7%	3.4%	7.5%	21.4%	61.0%	46.006 ***	0.193 ***
3	In the UK, thousands of male dairy calves are shot, and millions of male chicks are gassed annually, because they are worthless to the dairy and egg industries	12.7%	5.4%	10.4%	16.6%	54.9%	99.601 ***	0.280 ***
4	The British Dietetic Association has declared that 100% plant-based (vegan) diets can be at least as healthy as omnivorous diets	14.0%	13.5%	19.2%	18.1%	35.2%	73.793 ***	0.252 ***
5	Roughly 64 billion land animals are slaughtered annually worldwide (trillions including fish)	11.9%	17.6%	18.1%	20.4%	32.0%	77.137 ***	0.258 ***
6	More chickens exist than humans in the world	30.5%	14.7%	17.3%	16.0%	21.4%	22.348 *	0.139 *
7	Poultry are hung side-by-side by their feet as they proceed down a conveyor belt for stunning in a water-bath prior to death	26.2%	11.1%	13.0%	14.0%	35.8%	45.737 ***	0.199 ***
8	Mutilations of farm animals without pain relief (such as debeaking in chickens and tail docking in pigs) are common practices in the UK	25.2%	9.1%	10.9%	13.0%	41.8%	75.934 ***	0.256 ***
9	Farm animals live a fraction of their natural lifespan; for example, a cow can live up to 25 years, but is killed on average at 6 years	10.3%	11.1%	12.4%	20.7%	45.5%	58.766 ***	0.225 ***
10	Current levels of demand for animal products would be unsustainable without factory farming	7.3%	9.1%	14.5%	20.3%	48.8%	33.318 **	0.170 **
11	The UK has the potential to grow more protein crops than it currently does	17.6%	14.7%	16.8%	20.7%	30.2%	33.096 **	0.169 **

Notes: 1. The likelihood ratio chi-squared test was used for items 2-3; the Pearson chi-squared test was used for the other items. 2. Levels of significance: * *p* < 0.05, ** *p* < 0.01, *** *p* < 0.001.

**Table 8:** Dunn-Bonferroni post-hoc test on median values of the Agricultural Knowledge Scale between UK yoga teachers in different diet groups.

Diet type	Omnivore	Vegetarian	Plant-Based
Omnivore	-	Stat value -57.651 ** SE 15.000	Stat value -110.212 *** SE 12.921
**Vegetarian**	Stat value -57.651 ** SE 15.000	*-*	Stat value -52.561 ** SE 15.678
**Plant-based**	Stat value -110.212 *** SE 12.921	Stat value -52.561 ** SE 15.678	*-*

Note: Levels of significance: * *p* < 0.05, ** *p* < 0.01, *** *p* < 0.001.
